# Follow the ‘Ting: sorghum in South Africa

**DOI:** 10.1080/15528014.2021.1984631

**Published:** 2021-10-12

**Authors:** Laura M Pereira

**Affiliations:** a Centre for Food Policy, City University of London, UK; b School of Life Sciences, University of KwaZulu-Natal, Pietermaritzburg, South Africa; c Global Change Institute, University of the Witwatersrand, Johannesburg, South Africa

**Keywords:** Sorghum, South Africa, indigenous foods, follow the thing, food system, sustainability, cultural heritage, gastronomy

## Abstract

This paper follows sorghum, an indigenous, but currently underutilized, grain in South Africa, through six encounters to discover its potential to transform the country’s food system. By listening to stories from diverse perspectives, it shows that the re-inclusion of sorghum could not only diversify diets, but could also move toward breaking colonial stereotypes of what constitutes aspirational food. It employs a Follow the Thing method to unpack the multiple identities of sorghum and the role it could play in galvanizing a healthier, more diverse food system. By opening up to a radical following method that does not constrain the researcher, the underlying stories associated with sorghum are highlighted, which coincides with a shift in perception of the multiple potentialities that the crop embodies. The research highlights that a strong cultural link to sorghum remains in South Africa and that if innovation could be broadly interpreted, this might invigorate a richer engagement with sorghum, not just as a commodity, but as a culturally significant food.

## Introduction: Sorghum (English); graansorghum (Afrikaans); mabele (Sepedi, Sesotho, isiNdebele); amabele (isiZulu); amazimba (isiXhosa)

I work hard every day to make my beer
(Umqombothi)
Wake up early every morning
To please my people with African beer
(Umqombothi)
I make sure the fire burns to make my beer
(Umqombothi)
My special beer Umqombothi

These are the opening lines to a song by the famous South African singer, Yvonne Chaka Chaka. She is singing about making “umqombothi” or “utshwala;” a beer traditionally made from maize and sorghum by the family matriarch for special occasions, such as contacting the ancestors (*amadlozi*), the returning of boys from initiation (*abakwetha*), weddings, funerals and traditional meetings (*izimbizo*). There is a concern that as South African’s preferences shift to commercially brewed beers that the art of traditional brewing will become “*a dying art: People are just not interested in their history anymore and their [traditional] food. … We are losing the culture of food*” said Nomsa Khitwa, a traditional brewer from the Eastern Cape in an interview with Taylor (2017). As well as traditional beer, the Tswana people of South Africa also make a fermented porridge (Ting ya mabele) made from sorghum (See Sekwati-Monang and Gänzle 2011). It is now registered on Slow Food’s Ark of Taste; a collection of artisanal products that are steeped in culture, but that are also at risk of extinction as the traditional practices upon which they are based are lost or the species from which they are made become endangered.^1^ The loss of sorghum from the South African food system does not only have implications for climate adaptation and agro-biodiversity (Hadebe, Modi, and Mabhaudhi 2017), but is closely tied to nutrition security, cultural practices and a sense of identity (See Cocks, Vetter, and Wiersum 2018).

There is archeological evidence of the use of sorghum dating back 8000 years in the Sahara (Wendorf et al. 1992) and that the domestication of sorghum took place between Chad and Sudan (Wet 1977). Sorghum (*Sorghum bicolor*) formed an integral part of the caloric base of most Neolithic and Iron Age food-producing societies in sub-Saharan Africa (National Research Council 1996). It is a nutritious grain, with higher protein content than maize (National Research Council 1996). Although it has low protein digestibility due to its tannins, these have high antioxidant and anti-inflammatory properties, highlighting a trade-off between protein digestibility and its other significant health benefits (Awika and Rooney 2004; Cardoso Leandro de et al. 2017). Different ways of preparing sorghum can have different impacts on its nutritional properties, for example fermentation (as traditionally practiced across Africa) and extrusion cooking can have various nutritional benefits (Duodu, Minnaar, and Taylor 1999; Cardoso et al. 2014; Salazar-López et al. 2018).

Sorghum is highly variable with 28 subspecies of cultivated grain sorghums (all *Sorghum bicolor* var *bicolor*), 7 weedy species, and 13 wild species recognized (Snowden 1935; Wet and Harlan 1971; Wet 1977). The cultivated sorghums in South Africa are known as *Sorghum bicolor* (L.) Moench. They come in two varieties – a sweeter variety (GM) and a more bitter variety that contains more tannins (GH)- and in a range of colors, ranging from white through to red. It is mainly cultivated in the drier parts of South Africa and due to its drought tolerant features, it is often planted where conditions are such that other crops like maize might fail (See Rosenow et al. 1983; Taylor and Belton 2002). It is a fast growing, generally hot weather crop, but is adapted to a wider range of ecological conditions than perhaps any other food crop (National Research Council 1996). It is also adaptive to the projected impacts of climate change. Studies have shown that sorghum yields are likely to increase under CO_2_ enrichment in dry areas, but remain unaffected by higher rainfall areas (Ottman et al. 2001). Simulations in West Africa show that the traditional cultivars of millet and sorghum used by local farmers for centuries seem more resilient to future climate conditions than modern cultivars bred for their high yield potential (Sultan et al. 2013). The ease with which cultivated sorghums cross with wild species (such as *S. arundinaceum*) provides great scope for the plant breeder and “a vast range of genetic characters can be brought together in bewildering numbers of combinations” (National Research Council 1996, 142). The plant therefore holds much promise as a resilient option for farmers to plant under changing climatic conditions.

Extensive work on its production in South Africa under different environmental conditions further highlights this potential. Because rainfed agriculture constitutes more than 95 percent of agricultural land use in South Africa as a semi-arid country, water scarcity is a major limitation to production (Hadebe, Modi, and Mabhaudhi 2017). Different genotypes of sorghum have demonstrated adaptation to low water availability, making it a drought tolerant crop (Hadebe, Mabhaudhi, and Modi 2020b). Sorghum therefore uniquely fits production in water-scarce regions, due to its high and stable water-use efficiency, drought and heat tolerance, high germplasm variability, and comparative nutritional value (Hadebe, Modi, and Mabhaudhi 2017). Additionally, there is research on how different crop management strategies in the production of sweet sorghum can improve soil nutrient availability (Malobane et al. 2020) as well as management recommendations for its production in different agro-ecologies (Hadebe, Mabhaudhi, and Modi 2020a). However, despite its nutritional properties and resilience to climate change, it is socio-economically and geographically underutilized (Hadebe, Modi, and Mabhaudhi 2017).

Given these nutritional qualities, its bio-cultural significance, and environmental importance in the South African food system, why is it found less and less on our shelves? Could it have a role to play in a reconfiguration of a South African food system that is more closely tied to the land and its diverse cultures, rather than an increasingly homogenous, industrial and Westernized food system? South Africa’s food system is demonstrably unequal and increasingly unsustainable (Greenberg 2010; Satgar and Cherry 2020). Despite policy and civil society efforts, it continues not to be able to meet the needs of many of its citizens, with hunger sitting at a prevalence of 45.6 percent and still closely tied to race (Shisana et al. 2014). Various interventions have been put forward, such as the Policy on Food and Nutrition Security (Department of Agriculture Forestry and Fisheries (DAFF) 2013), but this has not led to any significant improvements in food security outcomes (Boatemaa, Drimie, and Pereira 2018). Recent proposals include the need to invest more strongly in local and indigenous crops that are more suitable to local environments, offer potentially better nutrition that processed grains and have the added benefit of cultural embeddedness and knowledge (Mabhaudhi et al. 2018). However, by virtue of many indigenous crops being neglected and underutilized species (NUS), there is very little information on which to build a case for their inclusion in policy recommendations (Akinola et al. 2020). The inclusion and promotion of sorghum as a crop in arid and semi-arid areas, especially among subsistence farmers, will improve water productivity and is expected to aid in food security, therefore meeting many food system needs (Hadebe, Modi, and Mabhaudhi 2017). However, despite the mounting evidence of its benefits, it continues to be a neglected crop. It is perhaps time for an alternative approach to unpack how sorghum fits into the complex South African food system by experiencing it from different perspectives across its value chain.

In this paper I ask the research question: What potential role does sorghum have in a more sustainable and equitable future food system in South Africa from the perspective of actors in its value chain? To do this, I aim to undertake an experiential exploration of sorghum through its value chain to unpack its diverse roles in a South African context. In doing so, I discover the various material realities that sorghum embodies-from cultural heritage through to commoditized crop- and learn to recognize its potential as well as its stigma. Being able to uncover such multiple identities is critical to this novel, more experiential approach. As such, it requires a research protocol that is sensitive to such plurality and that is sufficiently flexible to open up alternatives to the status quo. Instead of a conventional positivist epistemology, I instead opted to ‘follow the “*ting”* …

## Method

A conventional scientific approach of analyzing key informant interviews was too objective an approach to really being able to connect with and unpack the complex (Deleuzian) rhizomatic interlinkages of history, culture, environment, food, health and discrimination that I believe are embodied within sorghum. Again, there is not a lack of objective scientific research on sorghum production, but the aim of this project was to provide an alternative perspective that could open up new ways of understanding its diverse manifestations across its value chain. For this research to be effective in its goal to provide a new angle to sorghum’s potential in the South African food system, I needed an approach that could connect to the materiality of sorghum as it manifests in daily encounters. As it is our stories that shape us (Evans 2017), I turned to a strong narrative approach told from the perspective of the researcher as they uncover new perspectives through diverse encounters. This is a methodology of following where, as with all really good stories, “there is no real beginning or end-point,” but a rather a continuous journey of discovery that brings with it moments of clarity or enlightenment (Pereira 2010, 387). I wanted to experience sorghum as it was told to me by people who have a much closer connection to it than I, so that I might use these experiences to open new perspectives on the crop with implications for how it is perceived, regulated, grown, sold and bought in the future. It is therefore a very personal journey that, whilst as replicable as other ethnographic research, would not result in the same story. There is a well-established literature of similar endeavors to follow a specific food or crop; such as the work on bananas by Soluri (2005), apples by Legun (2015) and mushrooms by Tsing (2015). All of these require a level of trust that the narrator is being open and transparent in their subjective account. These works offer an inspiration to the exploratory journey that I undertake to come to terms with sorghum and its role in contemporary South Africa.

In this paper, I follow sorghum through the South African food system. According to Cook and Harrison (2007, 40), a “follow the thing” ethnographic approach allows for case studies where commodities and their biographies are “the organizing principles for post-disciplinary research” through which “diverse, different and surprising” relations can be identified whilst at the same “presenting evocative, engaging, affecting but jarring accounts of connected lives that readers can hopefully identify with and get wrapped up in.” By presenting this narrative account of following sorghum through the food system, I aim to build on this epistemology, and ultimately use it to elucidate informative, alternative ways to enable a more sustainable and healthy food system through the lens of indigenous foods.

The “follow the thing” method builds on multi-locale ethnographic research, which was initially inspired by a call for radical geographers to get beyond the veil of capitalist fetishism and unpack the sometimes disturbing connections between Western consumers and the distant strangers that produce these commodities (Cook 2004). My encounters begin with “*ting* ya mabele,” and so in this research I refer to following the “ting.” Whereas the method presented by Cook (2004) follows commodities through multiple countries, I have adapted this approach to trace the complex connections between consumption and production within a single country, South Africa, but a case study that arguably exhibits a similar context of inequality within its own borders. I encounter and then follow sorghum from 6 different starting points- and whilst these are in no way fully exhaustive, they are sufficient to illustrate the multiple identities of this specific food. Each of the following sections is inspired by the chapters in Tsing (2015) where she allows each new story of her Matsutake mushroom journey to reveal an important dimension to the political economy of the food. The end of each short encounter narrative leads to a question as a segue to the next encounter story. Each encounter is not exhaustive, but rather aims to provide sufficient information and context to appreciate that particular dimension of sorghum as experienced by myself.

The “encounters” with sorghum were documented and unpacked using a mixed methods ethnographic approach that used both standard qualitative interview techniques, as well as photography and experiential methods of cooking and eating, that provide the researcher with a research method of “get[ting] inside networks, go[ing] with the flows and look[ing] to connect” (Crang 2005 cited in Cook et al. 2006, 657) (Table 1 shows a list of all interviewees. Please note that where there is an Asterix next to a name, this is a pseudonym. Consent was obtained from all participants and the research was approved by City University Ethics committee, Soc-REC/80025567/22-04-18). These twists and turns make for a messy, but real, recounting of the stories of sorghum from different perspectives across South Africa. The encounters result in an investigation into the multiple identities of sorghum in the food system: “sorghum as cultural artefact,” “sorghum as agricultural product,” “sorghum as commodity,” “sorghum as research subject,” “sorghum as innovation” and “sorghum as gastronomy.” These diverse identities emerge throughout the story and the conclusions argue how opening up these alternatives is an important tool for strengthening the position of sorghum, and potentially other indigenous crops, in the South African food system.

## Limitations

This approach brings forth a plurality of perspectives and understandings, needed to innovate beyond business as usual. An objective, positivist approach would not have adequately captured such pluralism. Thus, instead of implementing a strict scientific method with the generation of a definitive set of data, analysis and results, I instead offer an exploration of alternatives as personally experienced from the position of the researcher. Such self-reflexivity of a researcher’s positionality is crucial when undertaking such work, especially in a development context (Kapoor 2004). The “results” should therefore not be read as objective facts, but rather as openings for more in-depth research (Law 2004). I make no claim to objectivity, but hope that the subjective outcomes of this following method still offer important insights for the field of food studies.

Further, there are also gaps in the encounters. These are due to both time and logistics factors as it would be impossible to undertake a truly exhaustive list of encounters due to the complexity of the food system. As the intention was to follow the sorghum value chain, certain actors, such as government officials, are not included in the study although they have a significant role to play in sorghum’s potential reinvigoration. These absences are noted, but considering there is already a lot of information from these sources in the literature, I decided that these perspectives were already well researched and did not require their own encounter. They are, however, referenced throughout. The aim in this paper is to use an experiential method to explore aspects of the sorghum value chain that are underrepresented in the literature. It provides some insights as to what interventions might be most usefully implemented where in the food system, in order to enable sorghum to play a more significant role in building a more sustainable, equitable and healthy food system. The stories also provide fine grain insights as to where there might be culturally embedded resistance to interventions. These insights emerge from the stories that are narrated, rather than from the analysis of raw data, and so should be interpreted as such.

## Results

### Sorghum as cultural artifact: Mabele for breakfast

**Figure 1:**
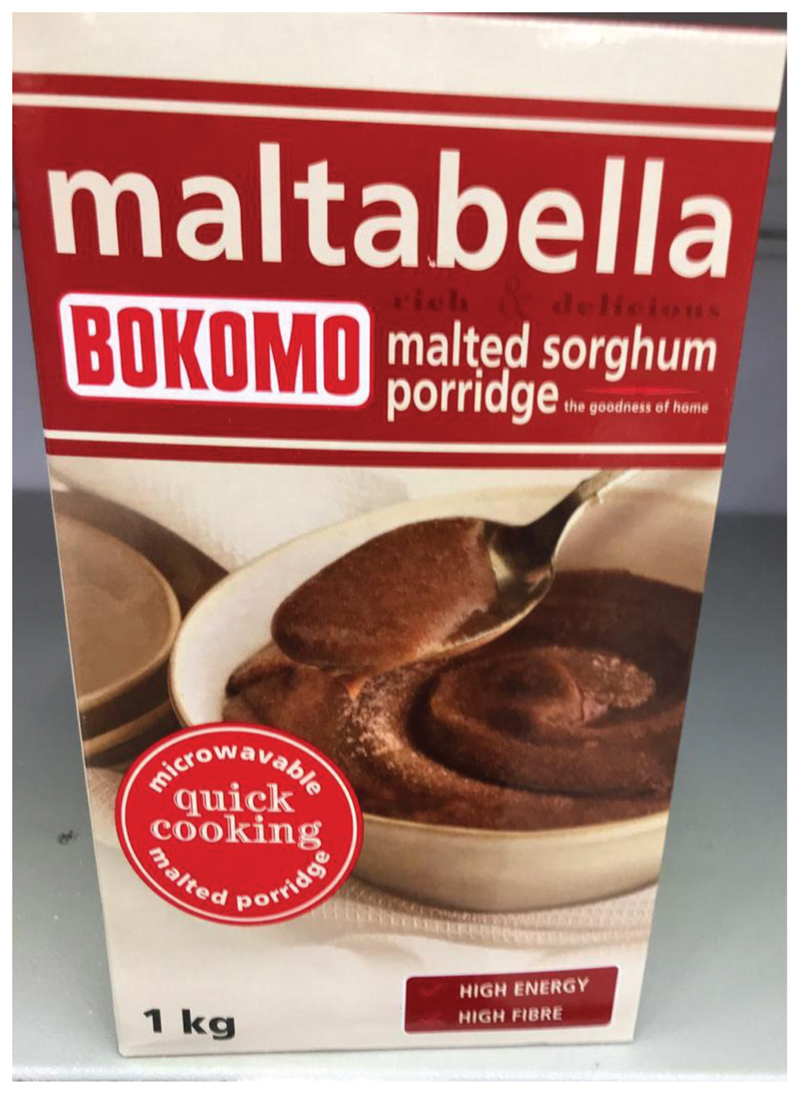
A box of Maltabella malted sorghum porridge (Source: Author’s Own).

My first memories of sorghum are of eating Maltabella porridge for breakfast (Figure 1). Bokomo^2^‘s Maltabella is made from 98 percent ground sorghum and, according to the South African online store in the U.K (Sanza.co.uk), it has been “a trusted brand for over 50 years” and some customers on the website have even lauded it with a poem, like this one by Joan Guyll:

Smooth and silky
Creamy and fine
Just the thing to make me shine
Milk or butter
Milk combined
Maltabella is devine (sic)!

The poem alludes to how the porridge is made, by sometimes adding milk and butter instead of just hot water. It is an iconic product for most South Africans, even if many of us have transitioned away from having it for breakfast. Considering my now distant relationship with sorghum as a breakfast cereal, when first needing to encounter sorghum, I contacted a friend and colleague, Malebogo, who grew up in Polowane, Limpopo Province. I know that she still tries to have sorghum for breakfast; she eats half a cup of sorghum (unground) every morning for breakfast, but will have Mabele (the porridge version) whenever she goes back to Polokwane to visit her parents. She grew up eating ‘ting ya mabele (fermented sorghum porridge) because it is a popular staple among her mother’s people, the Batswana, although they tend to have it for dinner rather than for breakfast.

Research on consumers in Polokwane, Limpopo, suggest that sorghum, which is easily purchased in town, is widely consumed, mainly as soft porridge, but also as thick porridge, fermented porridge and sorghum beer (Bichard et al. 2005). Factors influencing the consumption of sorghum include the age of consumers, the closeness of their ties to rural areas and their religion (Bichard et al. 2005). Sorghum is seen as being “healthy, nutritious and traditional, but inconvenient to cook and preserve” (Bichard et al. 2005, 125). Bichard et al. (2005, 140) found that a “need for tradition” among consumers underlies their demand for products derived from indigenous cereals. This demand is only partially satisfied in urban markets as there is 1-a lack of availability of whole sorghum seeds, and a range of flour textures (from very fine-Grade 1- to coarse-Grade 4); 2- no distinct different colors of sorghum grains (i.e., brown, red and white sorghum that is not mixed together) and, 3- no convenience foods such as ready-to-eat breakfast cereal, bread or biscuits made from sorghum that would appeal to the younger generation (Bichard et al. 2005).

**Figure 2:**
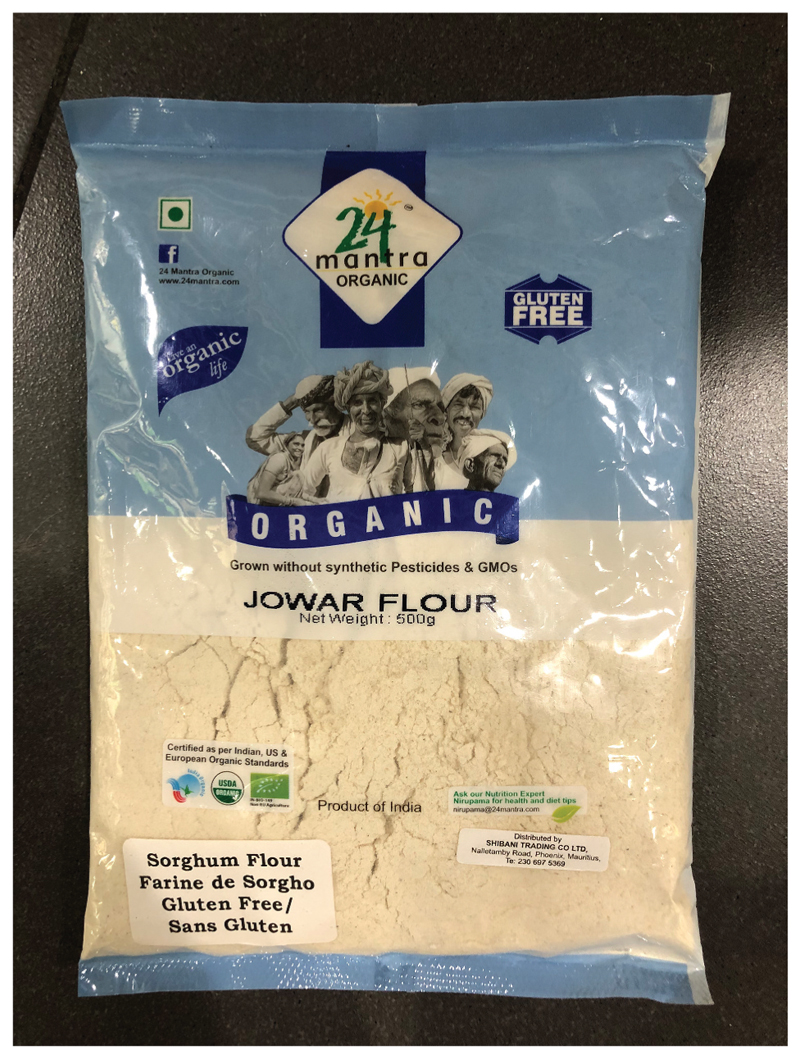
Ground sorghum from Wellness Warehouse, retailing at ZAR 40.95 (approximately 20 USD) per kilogram in December 2018 (Source: Author’s own).

What emerged strongly from Malebogo’s experience was the different accessibility of sorghum in the Western Cape versus Limpopo province. I remembered that we had once had to make a special excursion to find her breakfast sorghum in Cape Town. As far as she is aware, near where she lives, sorghum is only stocked by the Pick ‘n Pay super-market at Longbeach Mall in Noordhoek and that whole sorghum is even harder to find-she has only managed to locate it at Komati Foods and Nude Foods at an inflated price. At the time when Nature’s Choice^3^ stopped supplying whole sorghum, she even called them in an attempt to find out why they stopped supplying it. “Finely ground stone-milled sorghum” has subsequently re-appeared as a Nature’s Choice product and it can also be found on the shelves of Wellness Warehouse, although this sorghum is an organic certified flour (Jowar Flour) that is imported from India and retails at almost double the price of sorghum flour in conventional supermarkets (Figure 2).

But if sorghum is in demand in urban centers and commands a premium price, is this filtering back to the farmers who traditionally produced this grain?

### Sorghum as agricultural product: traditional farming

In Limpopo province, if small-scale farmers are able to grow sorghum successfully, they use it to make “brown pap” or mabele porridge. They tend to buy sorghum to make “Limpopo beer” or sorghum beer for traditional ceremonies rather than use what they cultivate in the field for this purpose. One of the big constraints to sorghum production in Limpopo, as well as the low productivity of seeds, is that according to farmers in rural areas, there is not that much demand for it.

We use maize as staple food and people making traditional beer, there aren’t as many-just a few people. Less demand is why (farmers) don’t cultivate too much, there is not that much assistance, not many policies. (*Temba*)

It can be difficult to keep track of how much sorghum is produced by smallholders as they do not take their produce to silo and mainly use it for their own consumption. In KwaZulu Natal province, sorghum is not produced as widely as it is in Limpopo, but there are a few farmers who are trying to bring the seed back under a programme for saving seed and valuing local varieties. This is not yet happening at a large scale, but in 5 community sites where the NGO Biowatch is working, in Umkhamyakude and Zululand districts, about 40 farmers have sorghum, but they are not yet marketing it. The farmers are still at the stage of multiplying seeds and making them accessible. One incentive for growing sorghum is to increase their crop diversity; some of the Biowatch farmers actually compete with each other in this regard, “*some of them grow (sorghum) because they want to reach a certain amount of diversity* … *some of them are keeping it to meet competitive targets*” (Lawrence).

A key challenge that these rural communities face is access to knowledge about how to store, process and consume sorghum.

Old people are no longer there to promote the eating part, people are just trying this and that, but there are people who last year have grown sorghum to get some more to sell … Some families that are not farmers will buy Maltabella from shops for porridge, and some of those that grow it will eat some, save some as seeds and send some grains to whoever wants it-i.e., sell it locally. (*Lawrence*)

Sorghum also needs certain techniques and technology for storage, and even de-shelling it requires special knowledge. Biowatch provides a platform for exchange and cross-fertilization of knowledge from elders, and experts who can train others in this so that the knowledge is passed on and used to make sure that no grains are lost.

One potential way forward is for farmers to form co-operatives, so that they can come together and approach the agriculture department for local level support as the extension officers “*don’t really have a clue about it*” (Lawrence). Although sorghum won’t ever compete with maize, even if farmers aren’t eating it, they will use it for brewing zulu beer (umquombothi). “*In our culture, every ritual needs zulu beer*” (Lawrence). He believes that if more sorghum were grown and made available, transformation into other products would happen naturally. This would also help to overcome the challenge of bird predation (Box 1).

Box 1 **Africa’s feathered locust.**

“*Amazimba needs a lot of labour and nowadays children go to school and there is no one to help in the fields and chase away the birds*” (Notes from a focus group with smallholder farmers in Pondoland region of the Eastern Cape, See Kennedy, 2015).

“*The big challenge is, even those producing it (sorghum), they have the problem of birds-It’s not a hybrid variety or commercial variety*” (Lawrence on smallscale farmers in KwaZulu-Natal). The Red-billed Quelea has been dubbed “Africa’s feathered locust” (Mundy and Jarvis 1989). The exposed grain of the sorghum plant makes it particularly vulnerable to bird predation and the quelea birds are notorious culprits. The birds prefer sweet sorghum, (which is also better for milling, food and bioethanol), so farmers have opted to grow bitter sorghum, in part to reduce predation by these birds. As acreage of sorghum has decreased, the relative amount of predation on plots has increased-the same number of birds predating on fewer crops means that the damage is considerably higher. If farmers were able to plant their sorghum together, rather than in isolated plots, this could help to alleviate the bird predation problem: “*There is potential if the farmers can come together and plant more, therefore the birds can eat some and leave some. It needs people to be told about that because they’re not aware-awareness and education is necessary*” (Lawrence).

“*We need better cultivars for sorghum* … *and knowledge*” (Sue*). Public awareness to educate people and get more information on the practices and market value of sorghum in communities that can help improve production are necessary interventions. Awareness raising on the benefits of sorghum is critical and can also work on related crops like pearl millet. “*It’s key to work with smallholder farmers to identify their priority traits, exploiting the genetic diversity of local traditional varieties, and developing new varieties based on their priorities*” (Dr Shargie from the Agricultural Research Council (ARC)). Combined with improved varieties, smallholders also need access to technical knowledge and extension officers that are familiar with sorghum (Botiabane et al. 2017).

Niche markets are opening-especially gluten free and organic, but in order to take advantage of this, smallholder farmers need help not only with production, but also with bringing their excess sorghum to market.

So, how viable is the commercial aspect of sorghum?

### Sorghum as commodity: the business of processing sorghum

**Figure 3:**
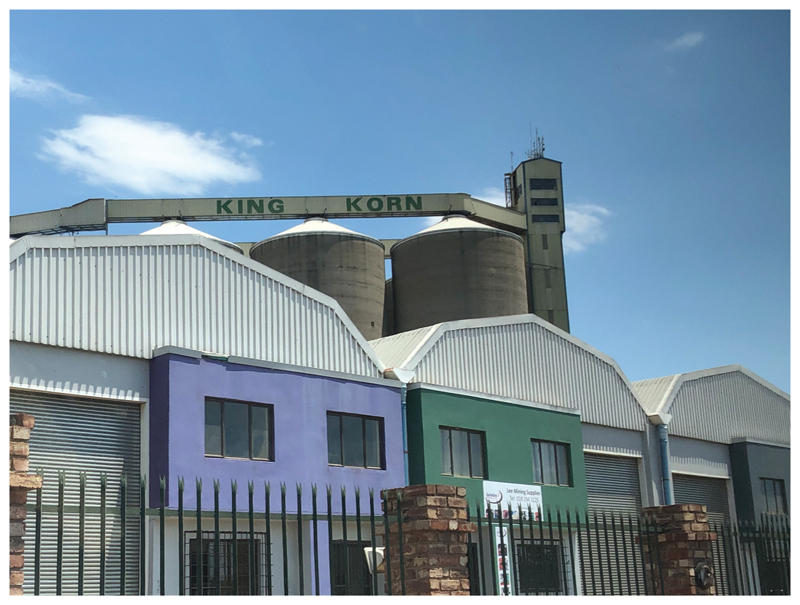
The towers of the King Korn processing plant of Tiger Brands in Potchefstroom (Source: Author’s Own).

In South Africa, the name most synonymous with sorghum food products is Tiger Brands. Around the corner from the Agricultural Research Council Grain Crops Institute in Potchefstroom is the main sorghum processing plant of Tiger Brands, home to King Food, one of the iconic brands associated with sorghum (Figure 3). Tiger Brands is the market leader in sorghum processing, but it’s a changing landscape just “*as Smirnoff* (vodka) *is replacing beer in traditional ceremonies*” (Sue*). King Food Corporation was established in 1922 by the late William Kirsch who started a sorghum malting plant at Potchefstroom in South Africa. The plant was one of the largest of its kind in the Southern Hemisphere, producing a range of sorghum-based products for the consumer market and specific industries.

**Figure 4:**
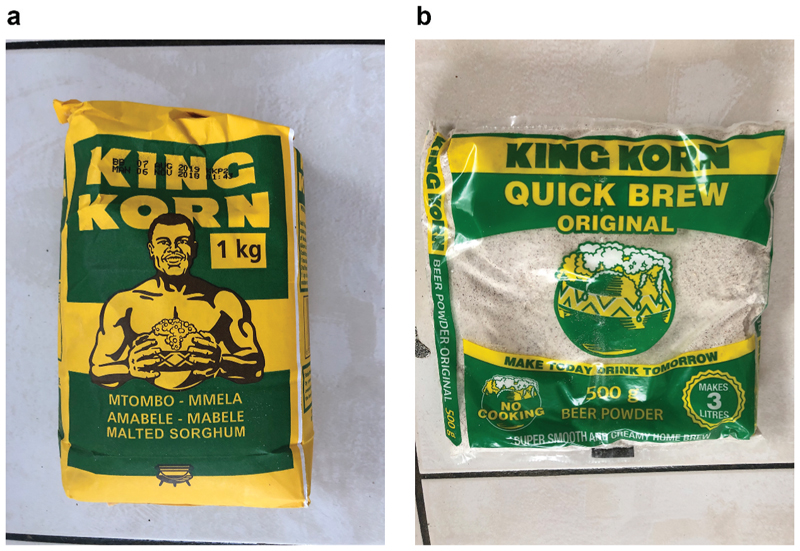
King Korn malted sorghum and quick brew original beer powder from Tiger Brands (Source: Author’s own).

Sorghum is once again close to the company’s heart as they shift toward a health and wellness focus that is trying to promote “the goodness of sorghum,” especially for current “non-users” (those that traditionally don’t have history of eating sorghum). With urbanization, people are looking for convenience and “*not spending 7 days to brew beer*,” and so the market has to respond (*Sue). The beer powder, an accelerator for the traditional brewing process is an example of this-it is combined with King Korn to speed up the traditional brewing process (Figure 4). An interesting side-note is that even though neither brewing product actually contains alcohol, the beer powder still incurs excise duty (usually only applied to petroleum, alcohol, tobacco and luxury items) making it more expensive.

Although sorghum beer is becoming less popular, it still has cultural significance across all LSM^4^ groups. Although the youth may not know how to brew the beer, some are still connected to the tradition and it is important to maintain this connection to culture. According to the Tiger Brands website:

Home-brewed beers are a part of South Africa’s heritage, as an important part of our weddings and funerals. King Korn is a traditional sorghum homebrew brand, and the number-one selling home-brew malt in South Africa. King Korn is a part of your lifestyle because it has a strong heritage that stands for tradition and Ubuntu. Our natural ingredients and consistent results have given us a reputation for reliability for producing a good quality brew. You know you can rely on us for the best result when your home brew matters the most.

**Figure 5:**
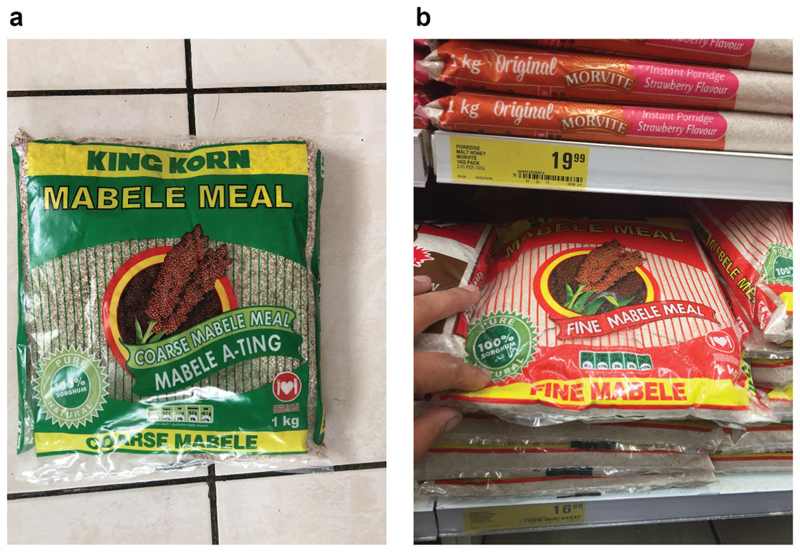
King Korn Mabele Meal (coarse and fine).

From the food side, Tiger Brands produces Mabele meal – either a soft porridge for breakfast or a stiff pap for dinner. These products include King Korn Mabele Coarse Meal, King Korn Mabele Fine Meal and the recent addition, King Korn Mabele Super Fine Meal “*for the more modern consumer*” (Figure 5). Morvite is the other main sorghum-based product of Tiger Brands. Morvite in the early days comprised predominantly of precooked maize and sorghum. It gained popularity in the South African mining industry where it was used as an instant energy drink by mine workers for mid-shift mine feeding (Phakathi 2017). It is hence commonly known today in this industry as “Phuza Amandla,” or “Drink for Power” (See Schilpzand et al. 2010). Its continued support was fostered by clinical trials conducted at the University of Potchefstroom in the 1990s, and research in mining accidents studied by Dr GD Campbell of the Themba Hospital in KaNgwane (*John). Morvite is used in child-feeding schemes as well as in nutritional studies on HIV infected patients (Botha 2007; Nzama and Napier 2017).

**Figure 6:**
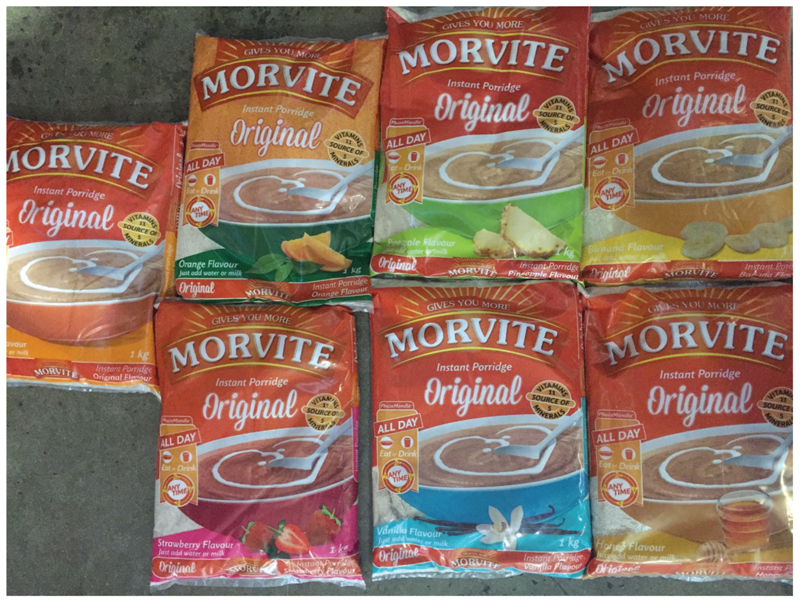
The 7 flavors of Morvite (Source: Author’s own).

According to John*, Morvite has undergone several formulation changes over the years to the purely sorghum-based product it is today. The sorghum used in the formulation is decorticated and puffed, which means that the product is “ready to eat” and requires no cooking by the consumer. It has a characteristic sweet-sour taste profile caused by the addition of citric acid to the formulation. Packed with 11 vitamins and 6 minerals, it is well formulated to meet the dietary requirements of school children, workers and sportsmen and it can be prepared as an energy drink with milk or water, or taken as a breakfast cereal. The presence of vitamins B1, B2 and Niacin act as coenzymes vital for oxidation, or burning of sugar to produce energy (released from the 77 percent carbohydrate that Morvite contains). Very importantly, the body starts utilizing the added sugar in Morvite within 5 minutes and will also suppress fat utilization keeping the body temperature and heart rate down. Consequently, the symptoms of fatigue and exhaustion disappear within 10 to 15 minutes after Morvite has been consumed. In 2004 Morvite was revamped with new packaging as well as the introduction of several new flavor variants in the market: it is currently available in Original, Strawberry, Vanilla, Banana, Honey, Pineapple and, most recently, Orange flavor (Figure 6). They come in package sizes of 1, 2, 25 kg and convenient 100 g and 150 g sachets mostly used by the mining industry.

it comes with marketing baggage where it is seen as a “porridge for the poor.” This association is taking it backwards, and is probably why people are not willing to pay more for it relative to maize, but it really is a brilliant product … it’s like Future Life^5^ on steroids. (Sue*)

**Figure 7:**
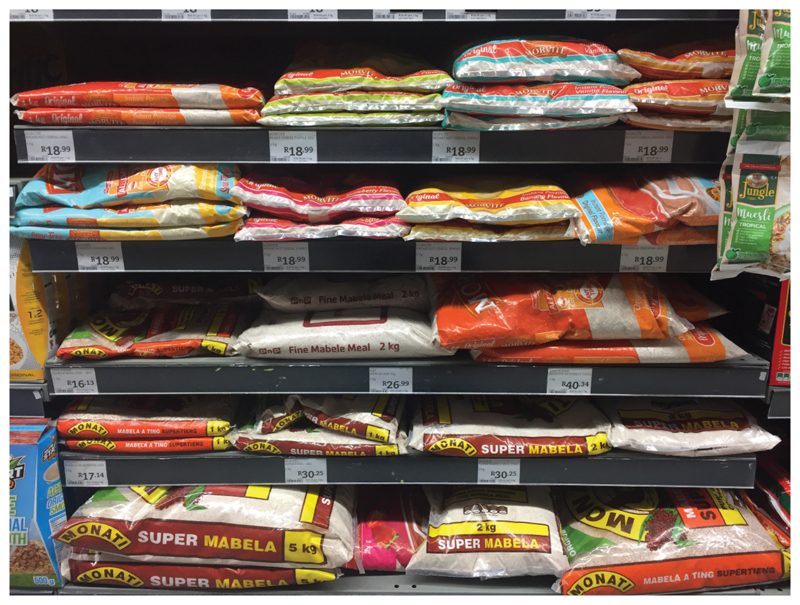
Packets of mabele meal and morvite on sale at a local supermarket (Source: Author’s own).

However, overcoming the stigma associated with Morvite, but also with sorghum more generally, requires a new marketing emphasis that includes a change in packaging. Morvite’s packaging is seen to be unattractive to some people, and its position in stacked packets, low on the shelf is also a problem according to *Sue, as foods sold in boxes rather than plastic bags are apparently perceived as better quality products (Figure 7). Furthermore, the health benefits of sorghum need to be made clearer to consumers as even lower LSMs are becoming health conscious around issues like diabetes. “*There is lots of work to do, but we will do it because we believe in it*” (Sue*).

One of the concerns for the industry is that at the moment, the use of sorghum appears to be expanding in some products, but only for the label of an additional “ancient grain,” i.e., into crackers. However, as it is not the main ingredient, this is not enough to sustain an industry. It is therefore necessary to look at how to expand into alternative products that are primarily based on sorghum, but that offer more innovative takes on conventional porridge. A potential organic market for sorghum would require broader logistics because if the factories remain the same, there is the potential for contamination from non-organic sources. Similarly, it is also difficult to make a 100 percent gluten free claim when using importing sorghum (as South Africa now does) because there is often trace of other flours in the shipments.

**Figure 8:**
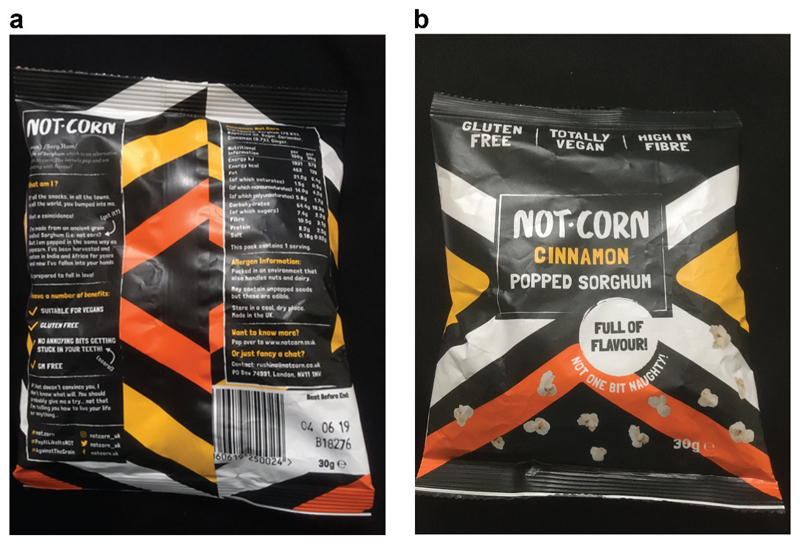
Not-corn popped sorghum on sale at Whole Foods for £1.20 a 30 g bag-that’s about the same price as a 2 kg bag of sorghum flour in South Africa (Source: Author’s own).

Internationally, sorghum seems to be taking the place of quinoa as a new superfood grain. In 2015, the Guardian called it the new “wonder grain” and in Australia is has been referred to as the new “super grain.^6^” A chance encounter in a UK supermarket found me sampling “Not-corn” popped sorghum, with the sorghum apparently being imported from Ethiopia (Figure 8). The popularity of sorghum and therefore its marketability is clearly spreading internationally. However, in South Africa in 2019, I could not find any interesting popular culture articles on the grain, although in 2018 Farmers Weekly magazine ran an article showcasing its benefits, especially given the looming threat of climate change and an even more arid farming environment for South African farmers.^7^ The potential of new markets for sorghum are clearly there, but from what could be gathered from key stakeholders, this potential is not yet being realized.

What is the kind of research and innovation that needs to be done to take advantage of this in the context of the South African market?

### Sorghum as research subject: So Yhum!

**Figure 9:**
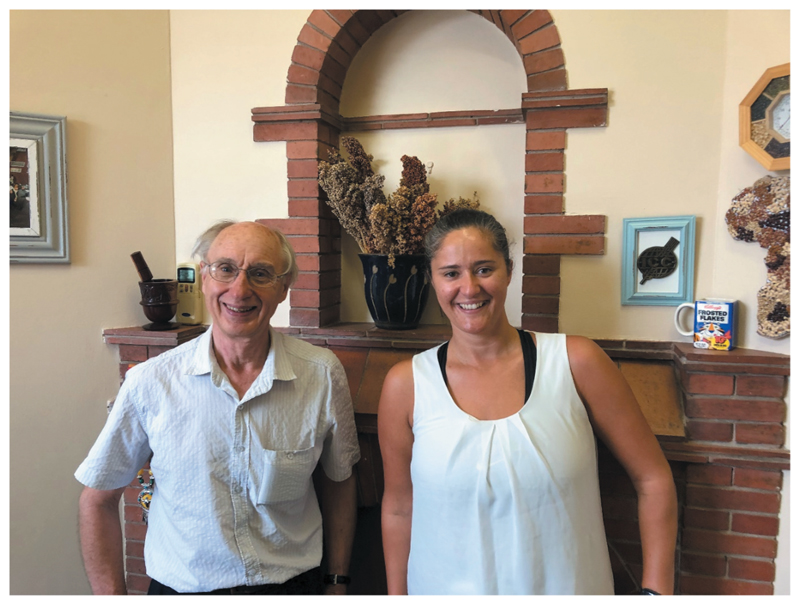
Prof John Taylor and I in his office, posing in front of different varieties of sorghum in the background (Source: Author’s own).

The University of Pretoria School of Food Sciences has been leading on the development of innovative sorghum products in South Africa. The driving force behind sorghum in the department is Prof John Taylor, who became head of the department in the 1990s focussing on food chemistry (Figure 9). He was specifically tasked by the South African government from the 1980s to work on sorghum and has become a world leader in the subject. Dr Henriette de Kock has worked with Prof Taylor over the years and her specialty is sensory science that analyses flavor, texture, aroma and how consumers perceive with their senses.

**Figure 10:**
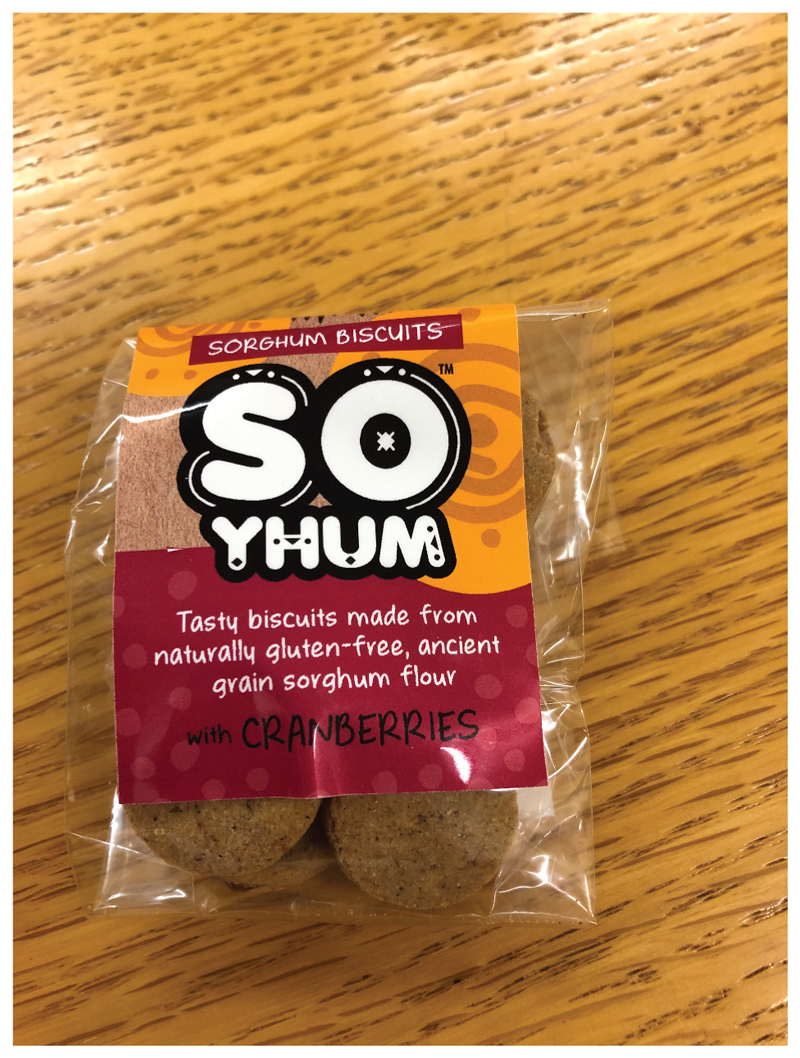
So Yhum Sorghum biscuits made at the University of Pretoria (Source: Author’s Own).

Although the department had constantly been developing modern food products from indigenous and “locally relevant” crops, this knowledge had remained in academic journals and had not actually made a difference for consumers. They had been developing sorghum-based biscuits for the past 10 years, initially for school feeding schemes in Kenya, but this research grant has now forced them to start a business. The intention of the project is to take graduates from Lesotho, Botswana and South Africa and to give them business skills to start a business based on this research. “*We’re now working on the ‘sorghum revolution’*” (Riette). Thus, So Yhum was born (Figure 10). “*I’m now ‘walking the talk’ of product development-let’s see what the future holds*” (Riette).

The product is actually designed to meet the needs of less affluent consumers, but they are not the targeted market, because

you need to create aspirational products. It shouldn’t be considered poor man’s food and there is a label-if you ask many people in SA about sorghum, they come up with two associations: beer and the “drunk uncle” and poor man’s food, “porridge”. (*Riette*)

They are trying to create modern products so there is novelty and interest in sorghum, especially for the new generation of urban dwellers who want convenience and modern products (See Bichard et al. 2005). Riette admits that sorghum has challenges as a food crop, but it is interesting to work with. She reiterated what had been raised by previous interviewees-that the biggest problems are that sorghum attracts VAT whereas maize and wheat are subsidized, its low yields compared to maize and bird predation. Its non-GMO status and drought tolerance are important promotional factors to promote with the crop gaining interest worldwide. As this continues to grow, it is likely that research development with result in improved yields. “*If there is market potential, hopefully there will be more production, but incentives like getting rid of VAT would help a lot*” (Riette). So Yhum currently sources their sorghum locally from a business entity that buys from commercial farmers, but Riette says that there is definite potential for small-scale farmers to enter the value chain. Although much of the investment with smallholders has been on producing sorghum for bioethanol, as the variety of sorghum (sweet-GM) is the same for food as it for bioethanol (Malobane et al. 2018), enabling these farmers to access food markets is critical. There is a lady, Tracee, who is selling sorghum products at the upmarket Bryanston market in northern Johannesburg, using sorghum that she sources from rural Limpopo women farmers. It is therefore clearly possible to build these value chains, but at the moment they appear to be ad hoc rather than systematic.

The consensus is that products like So Yhum probably need to be more widely available in the bigger supermarkets, although they are currently available in some niche stores. Awareness about sorghum again comes up as a key intervention that needs to be made in the system.

There needs to be an improved marketing and communication plan for sorghum. People need to access to more information so that they can make informed decisions in what they buy. People remember things-what grandmother would eat. There is a lot of marketing in the stories-it’s tradition. It’s gogo.^8^ (*Riette*)

Can niche markets sustain industries or do you need mass market buy-in? If the latter, is it possible to overcome centuries of stigmatization of indigenous African crops?

### Sorghum as innovation: gogo’s food, how to innovate our tradition

I grew up eating sorghum, not maizemeal, but fermented sorghum … Sorghum is: low GI, high fibre, gluten free, has anti-oxidants, non-haem iron, calcium, It’s non-GMO and drought resistant It has more protein It is plant based. (*Mpho*)

**Figure 11:**
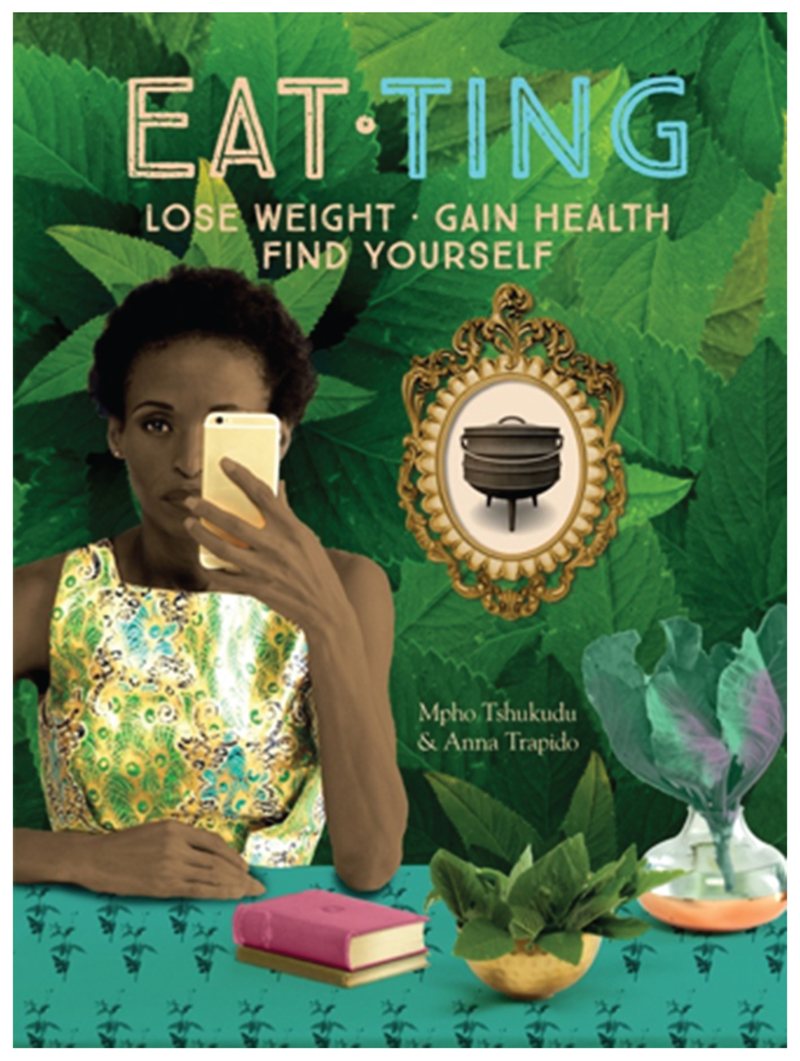
The cover of Mpho and Anna’s book entitled “Eat Ting.” As with the title of this paper, there is a pun on the word “Ting” that refers to fermented foods (Source: Quivertree publications).

Mpho Tshukudu is a strong advocate for reconnecting to what our grandmothers ate and revitalizing indigenous South African foods. Her acclaimed book “Eat ‘Ting” with food journalist Anna Trapido is a wealth of information on what healthy options South African indigenous foods and cooking offer (Figure 11). She is excited by the product innovation around sorghum, “*So many interesting things coming out of research. Sorghum cheese curls, edible spoons and straws made out of sorghum*!” (Mpho). She agrees with Riette that the big supermarket chains are where interventions should be made as it is the channel through which most sorghum is consumed, although she would probably prefer some of the other ingredients in the processed products also to be healthier, like having less sugar.

She also emphasizes the power of stories to shape people’s consumption choices: “*Most people have a positive story about sorghum-we need to tap into tradition and culture*” (Mpho). She described to me a conversation that she had with one of her clients:

Client: “*I stopped eating starch, but am still eating sorghum*”

Mpho: *“* … *but that’s starch*”

Client: “*But no, it’s not starch because my grandmother used to grow and eat it* … ”

There is a positive association of eating what our grandmothers would have eaten amongst high to middle income groups in South Africa. However, there is definitely a negative association for those who had to rely on it every day and cannot face it again “*now they’re in the suburbs, saying* “*I’ve moved on* … ” (Mpho). She says that the amaXhosa and amaZulu disparagingly refer to sorghum porridge as that “*brown pap from Pretoria*,” but that there remain stories about the powerful heritage of sorghum, even in these communities. According to legend, Shaka’s^9^ mother said not to give sorghum to the people because they would be happy and then couldn’t be controlled!

Mpho believes that science has now caught up with what the ancestors always knew about the benefits of sorghum and we can now provide evidence to back up why these plants are important (See National Research Council 1996; Awika and Rooney 2004; Awika et al. 2009; de Morais Cardoso et al. 2017). Much of sorghum’s increasing “superfood” status relates to its high antioxidant and anti-inflammatory properties that are attributed to its tannins (National Research Council 1996). However, overcoming historical stigmas is not easy. She argues that there is a need for a language to communicate the benefits of indigenous foods. There is no “*finish your thepe* (amaranth), *it will be good for your nails*” sayings as there are for foods like carrots. We need to develop these popular sayings to raise awareness about the benefits of eating these foods. She also mentions that chefs’ relationship with the heritage of local people could be a potential leverage point to shift perceptions of indigenous foods. However, this needs to be done with full recognition of the tradition and culture associated with these foods.

Are there any examples of chefs making use of sorghum in high-end restaurants and if so, what is their motivation?

### Sorghum as gastronomy: Wolfgat-the best restaurant in the world

My final encounter with sorghum takes me to Wolfgat restaurant in Paternoster-a month before it was crowned the best restaurant in the world.^10^ Owner and chef, Kobus van der Merwe, his partner Roelie, and a colleague and friend, Loubie Rusch from Making KOS and the NGO Local Wild, sat down and spoke to me about why they have been experimenting with putting sorghum back on people’s plates. It all comes back to that childhood classic, Maltabella …

Kobus: “*For me, the first connection with sorghum obviously was through Maltabella* … *malted sorghum porridge. That for me is a nostalgic thing* … *I mean growing up in the Northern Cape, it was very much mielie pap (traditional porridge made from maize), but I was more of a Maltabella kid*.”

Roelie: “*I just grew up on it (Maltabella), that’s why I love it-I never stopped using it, I just started experimenting more with it recently. I had it for porridge every morning*.”

Loubie: “*I also started with Maltabella. I’ve been playing a while with whole sorghum, but also with flour. When doing workshops, I want to introduce people to local grains so I use sorghum and millet*.”

**Figure 12:**
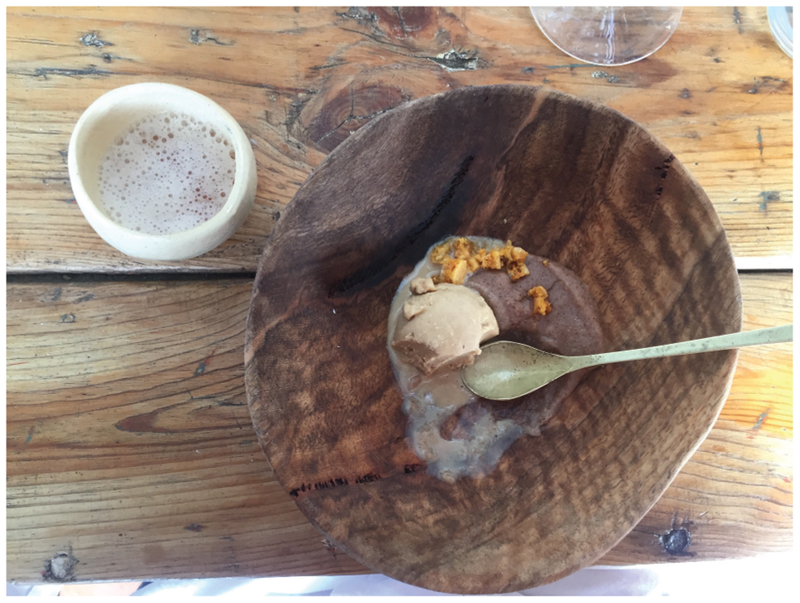
Wolfgat’s sorghum porridge and beer ice cream dessert (Source: Jenny Willis).

Sorghum is a relatively new addition to the winter menu at Wolfgat:

I’ve only recently started playing with sorghum at Wolfgat. All winter last year, we served a sorghum porridge as dessert. Not a malted sorghum, just a normal sorghum porridge adding a little bit of raw sugar, and a dash of cream-we served it with beer ice cream … people were blown away (Figure 12)! Whole kernel sorghum I cooked and then marinated in a sage ash with a bit of oil, so it actually became black and looked like caviar so we served it with fish … I’m dying to keep using it on the menu, but to me it feels more wintery. Unless you do a sorghum salad, but I dream about it more in a winter sense. (*Kobus*)

**Figure 13:**
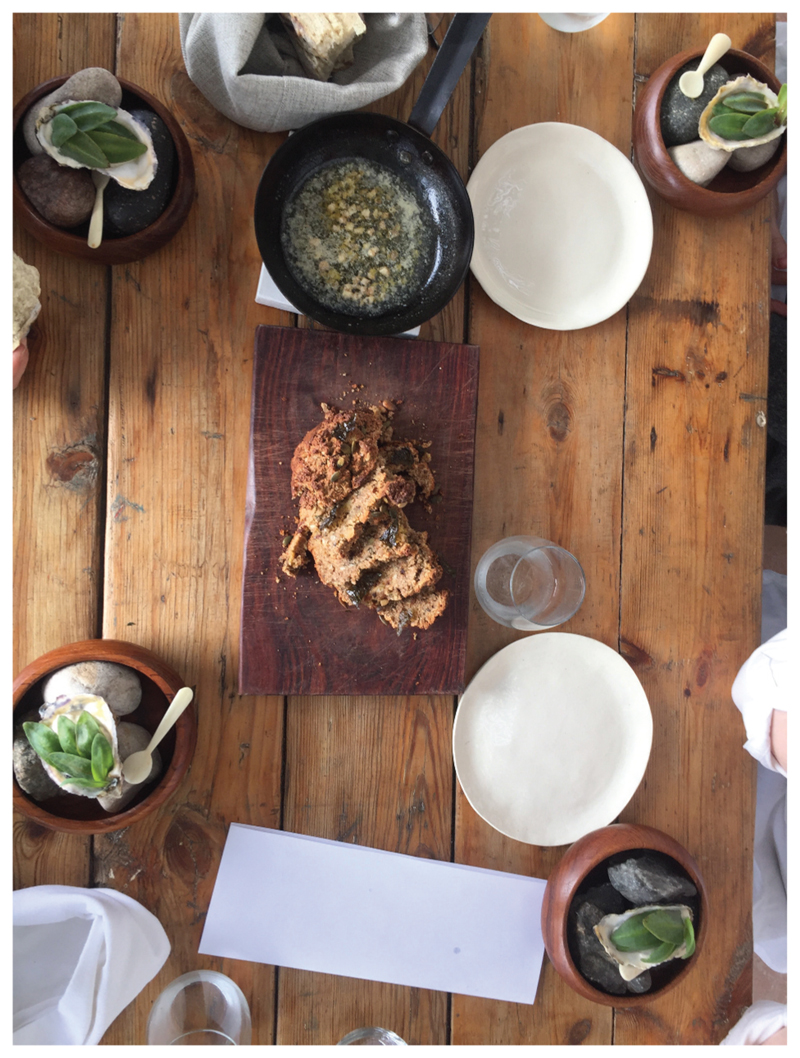
Sorghum bread on the table at Wolfgat (Source: Jenny Willis).

At Wolfgat, the most consistent use of sorghum is for their gluten free bread, which is made from sweet potato and sorghum and then flavored with seaweed (Figure 13). Sometimes they use almond flour, but as they can get people with nut allergies, sorghum is the main flour that goes in there. “*So that to me has been a revelation-people rave about that bread and they can’t believe it doesn’t have any gluten in it-it’s got a nice, nutty texture, like a seed loaf or a nice course country loaf*.” (Kobus). Loubie has also experimented with making crackers and crunchies with sorghum flour. As sorghum does not have gluten, there is very little stickiness to the dough and so in order to bake with, it needs something to bind it, like almond or chickpea flour. She has also prepared it more like a polenta, but instead of frying it, she puts it under the toasting machine and it goes really flat and forms a lattice- “*beautiful, crisp and light-We really haven’t yet started to push the boundaries on what sorghum can actually do*” (Loubie).

All of them source their sorghum from Yellow Submarine^11^ in Cape Town as they say it is really difficult to get it in ordinary supermarkets in the Western Cape, although further north in the country, it is more common-as Malebogo had already pointed out. Apparently mabele from a commercial supplier is not the same. When cooked,

it was much more glutenous and gooey … *Ja, it was yuk-I chucked it away. It looked like those starch projects they give kids in pre-primary. It had a funny translucency to it. I don’t know, it was weird, weird*. (*Kobus*)

Yellow Submarine’s sorghum, on the other hand, is great and is all locally sourced. They are now milling it into flour and have recently started stocking white sorghum as well as the more common red sorghum. It is the red sorghum that is mainly being used by the innovators, although they are keen to experiment with the white variety. They all concurred that it is difficult to access good quality sorghum unless “*you’re in the know*.” But most people who are into healthy eating know about Yellow Submarine that is based in Ottery, Cape Town and Komati Foods (as mentioned by Malebogo). Outside of these stores, in niche health food shops, it is very expensive and not very accessible to your average consumer. This was reinforced by my visit to Wellness Warehouse where they stock organic sorghum flour from India (See Figure 2). Increasing the number of suppliers and thereby lowering costs would be hugely beneficial for the average consumer.

**Figure 14:**
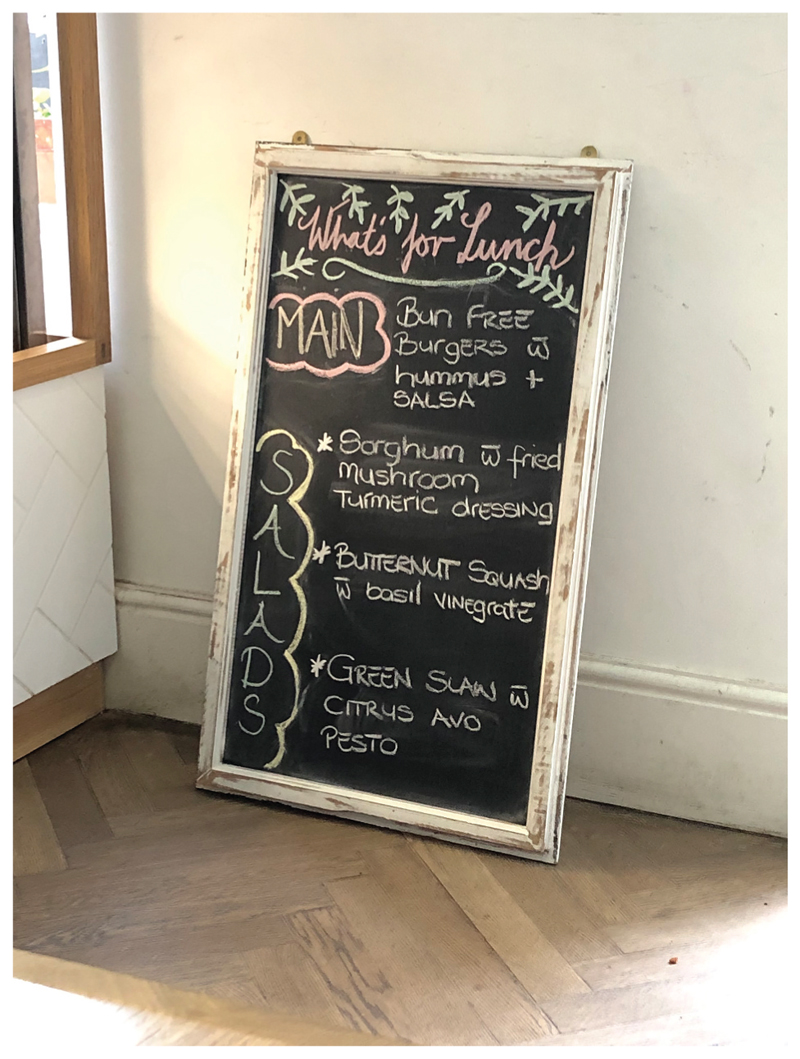
Sorghum salad on the menu for lunch at Spirit Café, a healthy vegetarian restaurant in Cape Town (Source: Author’s Own).

Sorghum innovation by people like Kobus, Mpho and Loubie is starting to trickle down to more conventional cafés and restaurants. I encountered a health-focused vegetarian restaurant in Cape Town that had sorghum salad on its menu (Figure 14).

### Discussion: sorghum’s multiple identities in the context of the South African food system

It is the role of critical geographic approaches to scrape away at problematics and to unpack constructed understandings of commodities in order to reveal the oft overlooked power of things (Pereira 2010). The aim of this kind of research is therefore not to posit solutions, but to cut through multiscalar politics by engaging with the “thing-power” (Bennett 2004) of commodities, like sorghum, in a way that recognizes it as a messy material that cannot be coerced into a single identity. What has emerged quite clearly for me in my following of sorghum is that it demonstrates a plurality of identities, encapsulated in both the material reality of the plant, but also in how humans have related to it, both now and in the past. I propose that these multiple identities- and there are undoubtedly more that the research did not necessarily encounter-each offer up a potential point for strengthening the role of sorghum in South Africa’s food system. As well as having characteristics of Spinoza’s *natura naturans*-a lively “materiality that is always in the process of reinventing itself” (Bennett 2004, 350), sorghum is not only dynamic in time, but holds multiple identities at the same time and in the same space, depending on its relational configurations. Recognizing these plural relational construction of identity-for example where sorghum in the form of mabele porridge on a plate in Wolfgat is at the same time gastronomy as well as being a cultural artifact-opens up many more options for how to engage with the crop than if one thinks of it in the conventional sense of being the same static commodity irrespective of context and relations.

At the same time, these multiple identities that are dependent on different contexts, histories and experiences make it that much more difficult to regulate or incorporate into public policy as an intervention to strengthen the South African food system. Conventional governance systems, for example in the development of public policy, operate with a very linear interpretation of the subjects that they wish to regulate and in so doing, I argue, often land up missing the bar. The case of local and indigenous foods is a good example of where this penchant for policy to have a formulaic response lands up not having the intended result. For example, innovation systems in Nigeria aimed at opening up markets for cassava had been in existence from the 1980s and culminated in the development of a cassava bread product that finally hit the market in 2012 with substantial government backing- and failed (Pereira 2017). One of the main reasons for this failure was a lack of recognition of the importance, not just of market access and financial incentives, but of the multiple relations that people have to the food they eat, especially when it is entangled with aspirational lifestyles, a colonial history and a stark urban-rural divide as is the case with cassava in Nigeria (Pereira 2017). On reflection, recognizing these multiple identities and how they are relationally constituted, is critically important in eliciting a particular change, whether through policy or other measures. South Africa’s National Policy on Food and Nutrition Security (DAFF (Department of Agriculture Forestry and Fisheries) 2013), whilst attempting to take a systems approach to problem of food security, fails to talk to the cultural aspects of why we eat certain foods. As such, even if fully implemented (which it has not been), it leaves gaps that allows the current food system configuration to remain largely unchallenged. The National Strategy for Indigenous Crops in South Africa (DAFF 2014) that has been in draft since 2014 also falls into the trap of recognizing the benefits of NUS in a more sustainable and healthy food system, but without taking into the multiple identities and histories that these foods have, which could both help and hinder their invigoration.

Appreciating sorghum’s plurality is not only necessary for designing more holistic policy, it also opens up more alternatives about what types of interventions are even possible. It is by opening up such alternatives that more radical and transformative pathways that take us away from the lock-ins of the status quo are able to emerge as possibilities (Leach, Scoones, and Stirling 2010). Without them, none of this potential can even be recognized, let alone experimented with. What also emerged from this research is the interdependence of different components of sorghum to each other-sorghum as research subject has the potential to improve its commercial value as a farm commodity as well as a healthy snack on retail shelves. The added value of sorghum as a gastronomic entity has the potential to connect smallholder farmers to markets that are willing to pay a premium price for good quality, locally sourced grains. These interconnections can only be made visible through a complex exploration of plural, co-existing identities.

These diverse encounters highlight that sorghum does indeed show remarkable potential for expansion in the South African market. Furthermore, the lessons from sorghum can be applied to other indigenous foods-such as amaranth and Bambara groundnut-that also show potential benefits if mainstreamed into food systems. Reconnecting with indigenous foods and reinvigorating their place on our tables in Africa is an important task in the quest for more sustainable and healthy food systems (IPES-Food 2016; Bioversity International 2017). It is an increasingly poignant argument that building up diversity will help to build resilience in the South African food system and can help enable it to deliver food and nutrition security (Chivenge et al. 2015; Mabhaudhi et al. 2018; Hadebe, Mabhaudhi, and Modi 2020b). However, the successful reinvigoration of these traditional foods requires a concomitant reconfiguration of the colonial narratives that have denigrated these foodstuffs as “backwards” and not modern in popular culture. What the stories articulated in this narrative account show, is that by following a product like sorghum, and exploring its diverse identities and how people relate to it in different ways, it is possible to see that there are a diverse set of options available for how to use sorghum as a starting point for re-invigorating a larger movement toward the incorporation of indigenous foods for a more sustainable and healthy food system. These interventions range from making sorghum a zero-rated VAT commodity in order for it to be more competitive with maize, through to building value chains directly to smallholder farmers whose combined production of sorghum could not only meet the needs of a growing niche market, but would also help with issues such as bird predation. Intentional marketing campaigns and innovation in the food processing sector, when combined with its health benefits and nostalgic stories about heritage, could help to open up the sorghum market beyond what has been already on our shelves for decades.

### Conclusion

This project set out to explore what potential role sorghum could play in a more sustainable and equitable future food system in South Africa. Given present food system challenges, it is imperative that we seek out alternatives to the business-as-usual approach, and so to answer this question, I used a less conventional follow the thing methodology to encounter sorghum in different guises. The results have illustrated that sorghum has a plurality of manifestations in the South African food system, and that it is critical to acknowledge these in policies seeking to increase its market presence.

In 2019, the South African government identified sorghum as a crop of interest and the Department of Science and Technology planned to conduct an impact study on sorghum to highlight the major issues that hamper the sorghum market. Hopefully, this will lead to better government understanding and support of the industry. What is clear from following sorghum in the South African food system is that it is an indigenous food with a rich and complex history. It also has the potential to meet nutritional needs as well as build resilience to environmental change in the South African food system. The reinclusion of sorghum as an alternative staple food in the country could not only diversify diets, but could also go some way toward breaking colonially embedded stereotypes of what constitutes aspirational food. However, to reach this potential, the cultural aspects of sorghum, as well as the variety of places within which it is reinvented and innovated need to be acknowledged. Sorghum could play a pivotal role in building a more inclusive food system in South Africa, but to succeed, its multiple identities must be recognized in any interventions that are proposed.

## Figures and Tables

**Table 1:** List of people interviewed during the research.

Name/Pseudonym	Stakeholder group
Malebogo Ngoepe	Consumer
Sue*	Industry
Nemera Shargie	Research
Bob*	Industry and commercial farmers
Mark*	Industry
John*	Industry
Kobus van der Merwe	Chef and innovator
Roelie van Heerden	Innovator
Loubie Rusch	Innovator
Mpho Tshukudu	Dietician
Riette de Kock	Research and innovation
Lawrence Makhapili	Civil society and smallholder farmer in Kwa-Zulu Natal Province
Temba Chauke	Smallholder farmer in Limpopo Province

## Notes

1
https://www.fondazioneslowfood.com/en/ark-of-taste-slow-food/ting-ya-mabele/

2 Bokomo Foods was originally established in 1922 by seven Western Cape farmers, and has developed into a key player across numerous categories in the South African food industry over the last several decades. It is best known for its breakfast cereals.

3 Nature’s Choice traces is a company that was established in 1985 as the retail arm of the Natural Health Foundation. On their website, their vision is to be the standard bearers and benchmark of an infinitely healthier way of living: “We strive tirelessly to harvest the basic purity in the way that it was intended, to produce foods that are nourishing, uncompromised and unerringly pure. It is our duty to respect this natural order and to work with the earth, as we are a part of it and our bodies are designed to derive their full potential from it.” (https://www.natureschoice.co.za/about/)

4 The Living Standards Measure or LSM is a marketing and research tool used in South Africa to classify standard of living and disposable income. It segments the population into ten deciles based on their relative means, with LSM 1 being the decile with the least means and 10 being the decile with the greatest means

5 Future Life is a high protein super food porridge in South Africa: https://futurelife.co.za/

6
https://www.goodfood.com.au/good-health/say-hello-to-the-new-super-grain–sorghum-20170228-gumwla

7
https://www.farmersweekly.co.za/farm-basics/how-to-crop/ever-considered-sorghum/

8 Gogo is grandmother in isiZulu, but is a common word used by most South Africans.

9 Shaka kaSenzangakhona or Shaka Zulu is a legendary Zulu king of whom many stories are told:

“He is Shaka the unshakeable,
Thunderer-while-sitting, son of Menzi.
He is the bird that preys on other birds,
The battle-axe that excels over other battle-axes.
He is the long-strided pursuer, son of Ndaba,
Who pursued the sun and the moon.
He is the great hubbub like the rocks of Nkandla
Where elephants take shelter
When the heavens frown…”
(translated by Ezekiel Mphahlele).

10 Wolfgat won best restaurant at the Inaugural World Restaurant Awards held in Paris on February 18, 2019. https://restaurantawards.world/restaurant-year-wolfgat

11
https://yellowsubmarinefoods.co.za/
